# Translating the international scientific spinal cord injury exercise guidelines into community and clinical practice guidelines: a Canadian evidence-informed resource

**DOI:** 10.1038/s41393-019-0410-1

**Published:** 2020-01-16

**Authors:** Femke Hoekstra, Christopher B. McBride, Jaimie Borisoff, Mary-Jo Fetterly, Spero Ginis, Amy E. Latimer-Cheung, Jasmin K. Ma, Jocelyn Maffin, Lorne Mah, Christopher R. West, Rhonda Willms, Kathleen A. Martin Ginis

**Affiliations:** 1 School of Health and Exercise Sciences, University of British Columbia, Kelowna, BC Canada; 2 International Collaboration on Repair Discoveries (ICORD), University of British Columbia, Vancouver, BC Canada; 3 Spinal Cord Injury BC, Vancouver, BC Canada; 4 Rehabilitation Engineering Design Laboratory, British Columbia Institute of Technology, Burnaby, BC Canada; 5 School of Kinesiology and Health Studies, Queen’s University, Kingston, ON Canada; 6 Arthritis Research Canada, Richmond, BC Canada; 7 Department of Physical Therapy, University of British Columbia, Vancouver, BC Canada; 8 Department of Cellular and Physiological Sciences, University of British Columbia, Kelowna, BC Canada; 9 Centre for Chronic Disease Prevention and Management, Southern Medical Program, University of British Columbia, Kelowna, Canada; 10 Department of Medicine, Division of Physical Medicine & Rehabilitation, University of British Columbia, Vancouver, BC Canada

**Keywords:** Public health, Preventive medicine, Patient education

## Abstract

**Study design:**

Knowledge translation (KT) study.

**Objectives:**

To demonstrate how to use systematic, community-engaged methods to (1) translate the international scientific spinal cord injury (SCI) exercise guidelines into community and clinical practice guidelines, and (2) develop supporting resources.

**Setting:**

Canada.

**Methods:**

An expert panel of SCI researchers and stakeholders translated the guidelines and developed a supporting resource, using a KT process guided by an adapted version of the Appraisal of Guidelines, Research and Evaluation (AGREE) II Instrument. Pilot tests with end-users were conducted throughout.

**Results:**

The panel recommended (1) the two scientific exercise guidelines be combined and presented in a single message titled “The Canadian SCI physical activity guidelines”; (2) development of an online supporting resource, with educational and motivational information presented in “layers” to address the needs and preferences of diverse end-users. The top layer presents and explains the Canadian SCI physical activity guidelines. The deeper layers include information on benefits, overcoming barriers, activity examples, safety tips, and links to existing resources. Interviews with adults with SCI (*n* = 8) and survey-data from end-users (*n* = 90) showed that the guidelines and supporting resource were perceived as clear, useful, and appropriate.

**Conclusion:**

Using community-engaged methods, the two scientific SCI exercise guidelines were combined into one single physical activity guideline message. This KT process provides a template for groups in other countries to translate the scientific SCI exercise guidelines to their local settings using a similar systematic, community-engaged approach.

**Sponsorship:**

Rick Hansen Institute; Social Sciences and Humanities Research Council of Canada.

## Introduction

A fundamental barrier to physical activity (PA) for people with spinal cord injury (SCI) is a lack of knowledge and resources about the amount and type of activity needed to achieve health and fitness benefits [1–3]. To address this gap, an international consortium utilized a systematic and evidence-based approach to develop two international scientific SCI exercise guidelines [4, 5]. The fitness guideline states that “*for cardiorespiratory fitness and muscle strength benefits, adults with a SCI should engage in at least 20* *min of moderate to vigorous intensity aerobic exercise two times per week AND three sets of strength exercises for each major functioning muscle group, at a moderate to vigorous intensity, two times per week*” [5]. The cardiometabolic health guideline states that *“for cardiometabolic health benefits, adults with a SCI are suggested to engage in at least 30* *min of moderate to vigorous intensity aerobic exercise three times per week*” [5].

Two separate guidelines were formulated to delineate the relative strength of the scientific evidence underpinning each guideline. Given the supporting evidence, the panel endorsed the fitness guideline with a “strong recommendation” and the cardiometabolic health guideline received a “conditional recommendation” [5]. Although this delineation is important to maintain the scientific integrity of the guidelines, it is not necessarily important to end-users of the guidelines.

Indeed, the guideline development panel recognized at least three challenges to implementing separate fitness and cardiometabolic health guidelines in community and clinical practice settings [5]. First, two guidelines may be confusing as users may not know which guideline to follow. Second, the word “exercise” might limit people’s thinking about opportunities to be physically active (e.g., restrict thinking to activities performed in a gym). Third, introducing a new cardiometabolic health guideline may overshadow the pre-existing fitness guideline [6], and the importance of exercising for fitness. Recognizing that these challenges could only be addressed by adapting the scientific evidence, the panel recommended distinguishing between “scientific SCI exercise guidelines” and “community and clinical practice guidelines” [5]. Specifically, the panel called for a community-engaged strategy to translate the scientific SCI exercise guidelines into guidelines that can be communicated to and used in community and clinical practice settings [5].

Community and clinical practice guidelines can address the basic informational needs regarding what types and amounts of PA people should do. However, to be maximally effective, guidelines should be presented along with evidence-informed resources that teach and encourage people with SCI how to achieve the guidelines [7, 8]. Such resources are unlikely to be implemented and used, however, unless they are tailored to local audiences and settings (i.e., presented in a manner that aligns with users’ needs and preferences) [7]. By engaging stakeholders throughout the guideline and resource development and adaptation processes [9–11], we can ensure that we address the preferences, concerns, and feedback from people living with SCI in a specific community. Adopting such a community-engaged approach will result in products that are meaningful for potential end-users [10, 12].

The translation of the SCI scientific exercise guidelines into community and clinical practice guidelines and the development of supporting resources should be undertaken with the same rigor as the development of the guidelines themselves [5]. The Appraisal of Guidelines Research and Evaluation II (AGREE II) instrument is useful for ensuring rigor and transparency in the clinical guideline development process [13]. An adapted version of AGREE II has been used to guide the development of evidence-informed messages and resources that support delivery of PA guidelines to the general and specific populations, including Canadians with SCI [14–16]. This process has not yet been applied to the new SCI scientific exercise guidelines.

Therefore, the purposes of this knowledge translation (KT) project were to demonstrate how to use systematic, community-engaged methods to (a) translate the scientific SCI exercise guidelines into community and clinical practice guidelines suitable for dissemination and implementation in the Canadian context, and (b) develop an evidence-informed supporting resource for adults with SCI living in Canada. By documenting the systematic processes for translating the SCI exercise guidelines to local settings and the development of supporting resources, this paper may serve as a template for similar KT processes in other settings.

## Methods

### Overview

**Fig. 1:**
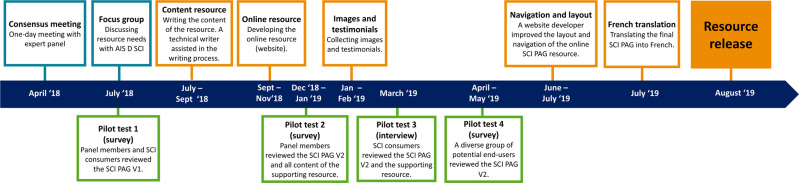
The timeline of the knowledge translation (KT) process of the scientific SCI exercise guidelines. This KT process included the translation of the guidelines and the development of an evidence-informed supporting resource. SCI PAG Spinal Cord Injury Physical Activity Guidelines.

An expert panel was established to help guide the KT of the scientific SCI exercise guidelines. Figure 1 provides an overview of the project timeline with key steps including a 1-day expert panel consensus meeting and pilot tests of the translated guidelines and supporting resource with potential end-users. Our steps were guided by an adapted version of AGREE II to ensure a rigorous, systematic, and transparent approach [13]. Appendix 1 describes the original and adapted AGREE II items and how they were applied in the current project. The AGREE II instrument consists of 23 items reflecting the following six domains: scope and purpose, stakeholder involvement, rigor of development, clarity of presentation, applicability, and editorial independence. This paper is organized following these six AGREE II domains and guided by the AGREE Reporting Checklist [17] (Appendix 2).

### Scope and purpose

The objectives, practical questions, target population, and potential end-users were formulated by the project leads (research lead: KMG; community lead: CBM). The expert panel members agreed upon the following statements:

- *Objectives*: To provide an evidence-informed resource that includes user-friendly information on the scientific SCI exercise guidelines and explains how adults with SCI can implement these guidelines into their lifestyle.
- *Practical questions:* What type of information is needed to support the dissemination and implementation of the SCI exercise guidelines in the Canadian community and clinical settings? How should this information be formatted and delivered to Canadians with SCI?
- *Target population*: Adults with SCI (all levels of injury up to C3) who are 18–64 years old and have been injured more than 1 year, including adults with SCI who are not yet meeting the PAG.
- *Potential end-users*: Adults with SCI, family members, friends and caregivers of adults with SCI, clinicians, practitioners, rehabilitation centres, community organizations, federal and provincial sport and health ministries, Canadian Society for Exercise Physiology, and public and private fitness centers.

### Stakeholder involvement

The expert panel consisted of SCI researchers, adults with SCI, a clinician, a KT specialist, and representatives of SCI community organizations. Appendix 3 describes panel members’ expertise and roles. Panel members attended a 1-day consensus meeting, reviewed drafts of the guidelines and the supporting resource, and provided several rounds of feedback. To ensure the guidelines and resource would be relevant to individuals with SCI who do not use wheelchairs, an additional focus group discussion was conducted to identify resource preferences and views of this group. Ultimately, stakeholder engagement processes resulted in feedback from nearly 100 adults with SCI and other end-users (e.g., healthcare and fitness professionals, community organizations) (Fig. 1).

### Rigor of development

#### Literature search

A purposive search was conducted to identify articles on (1) development and dissemination of PAG for people with SCI in a Canadian context, (2) PA participation, benefits, and predictors/correlates relevant for Canadians with SCI, and (3) development and adaptation of PAG messages. While a purposive search (instead of a systematic search) may introduce a risk of selection bias, it allows flexibility to include articles on topics relevant for our local context (i.e., Canada). Appendix 4 describes the criteria used to select articles for each of the above mentioned topics. Strengths of the evidence base included:

- the use of community-engaged methods to develop the previous SCI physical activity guideline, the previous Canadian PA resource, and the SCI exercise guidelines [5, 6, 14];
- the availability of Canadian-specific findings on PA predictors/correlates and preferences [18, 19], an overview of evidence-informed PA programs for people with SCI [20], and experimental evidence supporting the use of SCI-specific behavior change interventions;
- the availability of systematic reviews on what should be included in effective PAG messaging [7, 8].

Limitations of the evidence base included:

- insufficient evidence to support fitness or health benefits of PA for people with acute SCI [5, 21];
- insufficient evidence to draft specific recommendations for different types of activities (e.g., sports) [5, 21];
- a lack of SCI-specific systematic reviews on effective PAG messaging.

#### The multistep KT process

We used a multistep approach to translate the guidelines and develop a supporting resource:

- Panel members reviewed the scientific SCI exercise guidelines articles [4, 5, 22] and the previous Canadian PA resource [14] prior to the consensus meeting.
- Project lead (KMG) described the development of the scientific exercise guidelines at the beginning of the meeting.
- The panel discussed and made decisions regarding the key KT issues raised by the guideline development team [5] (how to message two guidelines, whether to use the term “exercise” or “PA”).
- In small working groups, panel members discussed and made recommendations for resource content (e.g., PA benefits, overcoming barriers). Working groups reported back to all members who provided further feedback.
- Three panel members (KMG, FH, SG) held a debriefing meeting to refine the information and prepare next steps.

Appendix 5 includes the agenda of the 1-day consensus meeting.

The panel recommended (1) combining the two scientific exercise guidelines into a single PAG message and (2) developing an online, mobile-friendly supporting resource, with educational and motivational information. Moreover, the panel recommended replacing the word “exercise” with “PA”, because PA is broader, including both recreational and daily physical activities. The panel decided to call the translated guidelines “The Canadian Spinal Cord Injury Physical Activity Guidelines (SCI PAG)”.

After the consensus meeting, two panel members (KMG, FH) worked with a graphic designer to draft the first version of the SCI PAG. The second and final versions incorporated the panel’s recommendations and panel members’ feedback. Appendices 6 and 7 outline key recommendations and supporting evidence for the Canadian SCI PAG resource. The first author (FH) created an outline of the complete supporting resource based on the panel members’ discussions during the consensus meeting. The MoSCoW method (Must have, Should have, Could have, and Won’t have), a prioritization method commonly applied in software engineering and business management, was used to prioritize panel members’ recommendations and suggestions [23]. In the context of this project, the first author (FH) labeled panel members’ recommendations and suggestions into one of the four MoSCoW categories based on discussions during the consensus meeting (see OSF [24]). The first author (FH) worked together with a technical writer to create the content of the resource.

Panel members reviewed the SCI PAG and the supporting resource and had the opportunity to provide feedback at three time points (Fig. 1):

- After drafting the first version (V1) of the SCI PAG (July 2018).
- After drafting the second version (V2) of the SCI PAG and a first version of the content of the supporting resource (January 2019).
- After drafting the third version of the SCI PAG (June 2019).

The first two rounds of feedback (pilot test 1 and pilot test 2) were collected via an online survey, and the last round of feedback was collected via email. The clarity, usability, and appropriateness of the SCI PAG and supporting resource were pilot tested at various stages of development with different groups of potential end-users (Fig. 1). Appendix 8 describes the pilot test procedures.

## Results

### Clarity of presentation

**Fig. 2:**
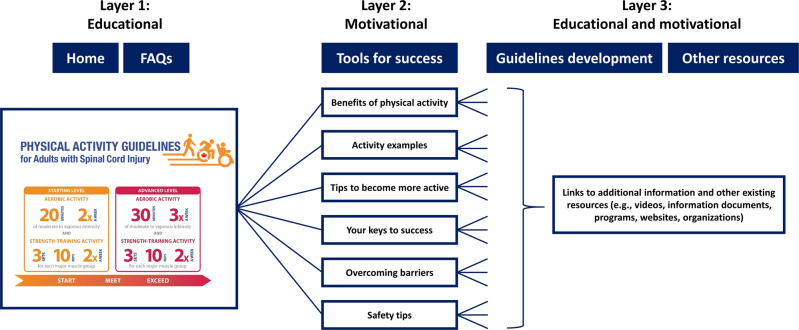
Outline of the different layers of the Canadian Spinal Cord Injury Physical Activity Guidelines resource. The resource is available via www.sciguidelines.com.

The panel recommended the development of an online resource in which the translated guidelines and supporting information is presented in “layers” to tailor the information to the needs and preferences of end-users (i.e., people who want more detailed information can go deeper into the “layers” of the website). Figure 2 depicts the layered structure of the Canadian SCI PAG resource.

#### Pilot test 1: panel members and adults with SCI

The clarity and appropriateness of the SCI PAG V1 were evaluated by expert panel members (*n* = 6). Panel members’ responses to survey questions were mixed with medians between 4.5 and 6.0 on 7-point Likert scales. Some panel members mentioned that the SCI PAG diagram was not clear enough and, in particular, not tailored towards adults with SCI who are not yet doing any PA. Additional telephone interviews with three adults with SCI confirmed that more information was needed to clarify the presentation and terminology used in the SCI PAG V1. Results are presented in Appendices 9 and 10.

#### Pilot test 2: panel members

The clarity, usability, and appropriateness of the SCI PAG V2 and the content of the supporting resource were evaluated by panel members (*n* = 9). Responses were positive with medians between 6.0 and 7.0 on 7-point Likert scales. Similarly, members were generally positive about the content of the supporting resource, illustrated by medians ≥ 6.0 or higher, with the exception of the items on risks of PA and information for power wheelchair users (Table 1; Appendix 11).

**Table 1:** Results of the expert panel survey (*n* = 9)—Pilot 2.

Survey questions	Range	Median (IQR)
*General objectives*		
• Is the resource appropriate for adults with SCI who are not doing any physical activity?	5–7	6.0 (1.0)
• Is the resource appropriate for adults with SCI who are doing some physical activity?	2–7	6.0 (2.0)
• Does the resource reflect the overarching message “*Start slow and gradually increase the amount of physical activity*”?	5–7	6.0 (1.0)
• Does the resource teach adults with SCI how to make smart and informed choices about being physically active?	4–7	6.0 (2.0)
• Does the resource encourage adults with SCI how to make smart and informed choices about being physically active?	3–7	6.0 (3.0)
*Physical Activity Guidelines Image*		
• Does the image (the diagram) provide a clear summary of the two levels of the Physical Activity Guidelines?	6–7	7.0 (1.0)
• Does the image provide clear instructions on the difference between the Starting Level and Advanced Level of the Physical Activity Guidelines?	6–7	7.0 (0.0)
*Physical Activity Guidelines—starting level*		
• Does the resource provide clear instructions about how much physical activity should be done in a week to receive fitness benefits?	6–7	7.0 (1.0)
• Does the resource provide clear instructions about the intensity level of physical activity to receive fitness benefits?	5–7	6.0 (1.0)
• Does the resource provide clear instructions about how much physical activity should be done in one session to receive fitness benefits?	5–7	6.0 (1.0)
*Physical Activity Guidelines—advanced level*		
• Does the resource provide clear instructions about how much physical activity should be done in a week to receive additional fitness and health benefits?	4–7	6.0 (1.0)
• Does the resource provide clear instructions about the intensity level of physical activity to receive additional fitness and health benefits?	6–7	6.0 (1.0)
• Does the resource provide clear instructions about how much physical activity should be done in one session to receive additional fitness and health benefits?	4–7	6.0 (1.0)
*Activity examples*		
Does the resource provide a variety of examples of moderate to heavy intensity activities that:		
• Help power wheelchair users meet the Physical Activity Guidelines?	3–6	5.0 (1.0)
• Help manual wheelchair users meet the Physical Activity Guidelines?	5–7	6.0 (1.0)
• Help people with ambulatory SCI meet the Physical Activity Guidelines?	4–7	6.0 (1.0)
*Benefits, overcoming barriers, safety tips*		
Does the resource highlight the most prominent evidence-based:		
• Benefits of physical activity for adults with SCI?	4–7	6.0 (1.0)
• Risks of activity/inactivity for adults with SCI?	2–7	5.5 (4.0)
• Barriers to physical activity for adults with SCI that are supplemented with feasible strategies?	4–7	6.0 (2.0)
*Strategies to promoting Physical Activity*		
• Does the resource provide adults with SCI a variety of ways to meet the Physical Activity Guidelines?	5–7	6.0 (2.0)
*Resources*		
• Does the resource include instructions for people with SCI when and how to contact the provincial SCI organization?	6–7	7.0 (1.0)
• Does the resource include relevant links to existing resources for people with SCI?	6–7	7.0 (1.0)
*Presentation and language*		
• Is the information in the resource presented in an appropriate and encouraging way?	6–7	6.0 (1.0)
• Is the tailoring of information in the resource appropriate for adults with SCI who are not doing any physical activity?	6–7	6.0 (1.0)
• Is the tailoring of information in the resource appropriate for adults with SCI who are doing some physical activity?	5–7	6.0 (2.0)
• Language—does the resource use language that is appropriate for adults with SCI who are not doing any physical activity?	5–7	6.0 (1.0)
• Language—does the resource use language that is appropriate for adults with SCI who are doing some physical activity?	4–7	6.0 (1.0)
*Logo*		
• Does the resource include an appropriate logo (i.e., the logo at the top of the document)?	4–7	6.0 (3.0)

Questions were answered on a 7-point Likert scale in which a higher response indicates a more positive response

#### Pilot test 3: adults with SCI

The clarity, usability, and appropriateness of the SCI PAG V2 and supporting resource were evaluated by eight adults with SCI. As illustrated in Appendix 12, participants were positive about the clarity, usability, and appropriateness of the SCI PAG V2 and the supporting resource. Participants provided specific directions to further improve the SCI PAG and supporting resource in terms of content, layout, and navigation (Appendix 12).

#### Pilot test 4: potential end-users

The clarity, usability and appropriateness of SCI PAG V2 were evaluated by a diverse group of potential end-users (*n* = 90) including people with SCI, family members/caregivers/friends of people with SCI, healthcare and fitness professionals, and representatives of SCI community organizations via a short online survey. All survey item medians were 6.0 or 7.0 indicating that the SCI PAG were perceived as clear, useful, and appropriate for adults with SCI (Table 2).

**Table 2:** Results of survey completed by potential end-users (*n* = 90)—Pilot 4.

Participants characteristics	Total		SCI end-users		Other end-users	
Age, mean (SD)	35.6 (11.8)		35.9 (12.9)		35.0 (9.8)	
Gender (% female)	37.8 (*n* = 34)		28.1 (*n* = 16)		54.5 (*n* = 18)	
Mode of mobility (%)						
Manual chair users only			47.4 (*n* = 27)			
Power chair users only			19.3 (*n* = 11)			
Manual and power chair users			26.3 (*n* = 15)			
Independent walker			7.0 (*n* = 4)			
Roles (%)						
Healthcare professional^a^					57.6 (*n* = 19)	
Recreation or fitness professional					30.3 (*n* = 10)	
Friends, family or caregiver					27.3 (*n* = 9)	
Representative of community organizations					12.1 (*n* = 4)	
Other (physiotherapist student, volunteer with SCI clients, SCI researchers)					21.2 (*n* = 7)	

Responses to survey questions	Total		SCI end-users		Other end-users	
	*N*	Median (IQR)	*N*	Median (IQR)	*N*	Median (IQR)
*Physical Activity Guidelines Image*						
• The image (the diagram) provides a clear summary of the two levels of the Physical Activity Guidelines.	89	6.0 (2.0)	56	6.0 (1.0)	33	6.0 (2.0)
• The image provides clear instructions on the difference between the Starting Level and Advanced Level of the Physical Activity Guidelines.	88	6.0 (2.0)	55	6.0 (2.0)	33	6.0 (2.0)
*Physical Activity Guidelines—Starting level*						
• The resource provides clear instructions about how much physical activity should be done in a week to receive fitness benefits.	90	6.0 (2.0)	57	6.0 (1.0)	33	7.0 (1.0)
• The resource provides clear instructions about the intensity level of physical activity required to receive fitness benefits.	90	6.0 (2.0)	57	6.0 (2.0)	33	6.0 (2.0)
• The resource provides clear instructions about how much physical activity should be done in one session to receive fitness benefits.	89	6.0 (1.0)	56	6.0 (1.0)	33	6.0 (1.0)
*Physical Activity Guidelines—Advanced level*						
• The resource provides clear instructions about how much physical activity should be done in a week to receive fitness and health benefits.	90	6.0 (2.0)	57	6.0 (1.0)	33	7.0 (1.0)
• Does the resource provide clear instructions about the intensity level of physical activity to receive additional fitness and health benefits.	89	6.0 (2.0)	56	6.0 (2.0)	33	6.0 (2.0)
• The resource provides clear instructions about how much physical activity should be done in one session to receive fitness and health benefits.	89	6.0 (2.0)	56	6.0 (1.0)	33	6.0 (2.0)
*Presentation and language*						
• The information in the resource is presented in a way that is interesting to look at.	90	6.0 (1.0)	57	6.0 (1.0)	33	6.0 (1.0)
• The resource is appropriate for adults with SCI who want to become more physically active but who are not yet doing any physical activity.	89	6.0 (1.0)	56	6.0 (1.0)	33	6.0 (2.0)
• The resource is appropriate for adults with SCI who want to become more active and who are already doing some physical activity.	89	6.0 (1.0)	56	6.0 (1.0)	33	6.0 (2.0)
*Usability*						
• The resource provides useful information for adults with SCI who are not yet doing any physical activity.	89	6.0 (1.0)	57	6.0 (1.0)	32	6.0 (2.0)
• The resource provides useful information for adults with SCI who are already doing some physical activity.	88	6.0 (1.0)	56	6.0 (1.0)	32	6.0 (1.0)
• The resource provides useful information for healthcare providers and fitness professionals.	88	6.0 (1.0)	56	6.0 (1.0)	32	6.0 (1.0)
• I will use this resource.	86	6.0 (1.0)	54	6.0 (1.0)	32	6.0 (1.0)
*Logo*						
• The logo is appropriate (i.e., the logo at the top of the document).	88	6.0 (1.0)	55	6.0 (1.0)	33	6.0 (1.0)
*Postcard idea*						
• How important is it to invest time and money in creating a paper version of the guidelines (postcard format)?	88	6.0 (1.0)	56	6.0 (1.0)	32	6.0 (1.0)

Survey questions are answered on a 7-point Likert scale, in which a higher response indicates a more positive response ^a^Healthcare professionals include medical doctors, physiotherapists, and occupational therapists. Some participants had more than one role. The variation in participants’ roles as well as participants’ mode of mobility illustrate the inclusion of a diverse sample of potential end-users

Details on how we addressed feedback from each pilot test, including the different versions of the Canadian SCI PAG, are available via OSF [24]. The final resource is available via www.sciguidelines.com.

## Discussion

This paper documents the use of systematic and community-engaged methods to translate the international scientific SCI exercise guidelines into SCI PAG suitable for dissemination and implementation in Canadian community and clinical practice settings. In response to stakeholder needs and preferences, the Canadian SCI PAG has two levels: the starting level is the SCI scientific fitness guideline and the advanced level combines the SCI scientific fitness and cardiometabolic health guidelines (Appendix 13). We also developed an evidence-informed supporting resource to help Canadians with SCI achieve the PAG.

The steps taken in our KT project are built upon the general literature on developing, updating, and adapting guidelines [10, 13, 25, 26], KT literature [9, 12, 27, 28], PAG messaging literature [7, 8, 11], and SCI-specific PAG literature [5]. Table 3 summarizes our 10-step KT approach by providing a general description of each step, an example from our KT project, and the supporting evidence. Our 10-step approach can serve as a template for other groups in other settings and other countries to translate the scientific SCI exercise guidelines to their local context. We believe that this 10-step approach will result in the development of high-quality, evidence-informed resources that are relevant and useful for end-users, and, ultimately, will have the potential to benefit local SCI communities world-wide.

**Table 3:** A 10-step evidence-informed approach to translate the scientific SCI exercise guidelines to local settings.

Steps	Description and examples	Supporting evidence
(1) Establish an expert panel	• Ensure the panel represents a diverse group of researchers and potential end-users (e.g., people with SCI, healthcare professionals, SCI community organizations). • Example: Appendix 2 describes panel members’ expertise.	[10, 28]
(2) Prepare an expert panel consensus meeting	• Use the AGREE II instrument as a preparation guide to ensure a systematic, rigorous, and transparent approach. • Example: Appendix 1 outlines the use of AGREE II instrument.	[13, 16]
(3) Discuss and create consensus on how to translate the SCI exercise guidelines	• Ensure all panel members are informed about the evidence base of the scientific SCI exercise guidelines. • Discuss how to translate the guidelines and this information should be formatted (type of resource) and delivered to the local audience. • Ensure all perspectives are heard when creating consensus on the translated guidelines and supporting resources. • Example: Appendix 4 includes the agenda of the meeting and OSF provides additional details on our consensus meeting.	[26, 28]
(4) Translate the SCI exercise guidelines to community and clinical practice guidelines	• Provide details on decisions made to draft the guidelines • Include descriptions on how the community and clinical practice guidelines are linked to the scientific evidence. • Example: Our OSF provides different versions of the SCI PAG.	[9, 10, 27, 28]
(5) Develop supporting resource(s)	• Ensure the resource(s) includes both *educational information* (how much PA should people be doing) and *motivational information* (why and how people should do PA). • Ensure the resource provides evidence-informed information. • Example: Fig. 2 outlines key topics of the Canadian SCI PAG resource, and Appendices 6 and 7 include links to the supporting evidence.	[7, 8]
(6) Pilot test the guidelines and supporting resource and address participants’ feedback	• Conduct pilot tests at different stages of development with panel members and other groups of potential end-users using a combination of quantitative and qualitative methods. • Ensure panel members approve final versions of the guidelines and supporting resource(s) before its official release. • Examples: Tables 1-2 include example questions of the different pilot tests conducted at different stages of the process.	[9, 10, 26]
(7) Disseminate the translated guidelines and supporting resource(s)	• Use a variety of strategies to disseminate the guidelines and supporting resource to different community and clinical settings. • Example strategies: social media, webinars, dissemination via panel members’ network, community organizations newsletters, clinical practice organizations.	[9–11, 25, 26]
(8) Monitor and/or evaluate its use in community and clinical practice	• Make a plan to monitor and/or evaluate the use of the guidelines and resource in community and clinical practice settings. • Example monitoring strategies: Google analytics, number of downloads, number of paper copies.	
(9) Share and publish the translation process *(optional)*	• Describe and share the translation process via academic paper(s).	Example papers: [14–16]
(10) Maintain relationships with panel members	• Maintain relationships with panel members to facilitate future community-engaged projects.	[30]

*AGREE II* Appraisal of Guidelines, Research and Evaluation, *PA* physical activity, *SCI PAG* Spinal Cord Injury Physical Activity Guidelines

### Applicability

#### Barriers and facilitators

The panel discussed a variety of potential barriers and facilitators to the dissemination and implementation of the Canadian SCI PAG and supporting resource. Most of the discussed barriers and facilitators were described in previous literature and/or identified and described by the scientific SCI exercise guidelines panel (e.g., limited time for clinicians to discuss PA, limited equipment availability for people with SCI) [1, 3, 5]. Potential barriers specifically relevant to the Canadian SCI PAG (resource) include: (1) people may get confused between the previous Canadian resource [14] and the new resource, (2) the amount of PA that people should be doing according to the SCI PAG might overwhelm people with SCI who are not yet doing any PA, and (3) it will be hard to reach adults with SCI who are not registered with one of the provincial SCI community organizations.

Potential facilitators to overcome these barriers include: (1) we have well-established partnerships between academic researchers and non-academic partners (e.g., people with SCI, community organizations, healthcare and fitness professionals) to optimize the implementation of the new SCI PAG resource; (2) the layered structure of the resource tailors the information to different needs of end-users (e.g., special information for people who are not yet doing any PA), and (3) we plan to promote the SCI PAG resource using different strategies (e.g., social media, presentations, webinars, word-of-mouth) to reach as many end-users as possible.

#### Implications

Potential implications include increased knowledge and awareness of the SCI PAG among Canadians with SCI, healthcare and fitness professionals, and policymakers, which may help Canadians with SCI to become (more) active, and may help healthcare professionals to discuss and prescribe PA in clinical settings. The SCI PAG resource may also help policymakers to formulate policies on improving PA opportunities for Canadians with SCI. Our SCI PAG resource may also have implications for SCI communities outside Canada. We hope that our efforts to KT the scientific SCI exercise guidelines will inspire other groups in other countries to develop similar resources tailored to their local context, and ultimately, positively impact people with SCI around the world.

#### Dissemination and implementation

The SCI Action Canada lab (www.sciactioncanada.ca) launched the Canadian SCI PAG resource in August 2019. A variety of dissemination strategies are being used to further promote its uptake in different Canadian settings. Strategies include, but are not limited to the dissemination via panel members’ personal networks, social media posts, communications platforms of SCI community organizations, communication via key SCI stakeholders (e.g., SCI fitness centres, rehabilitation centers, Spinal Cord Injury Research Evidence), webinars, and conference and community presentations. Furthermore, the panel suggested disseminating the SCI PAG resource via training programs for clinicians, fitness professionals, recreational therapists, and other health professionals and through collaborations with the Canadian Society for Exercise Physiology and other rehabilitation and fitness-related organizations.

#### Monitoring its uptake

We will monitor the number of visits and downloads of the Canadian SCI PAG resource via Google analytics. A simple evaluation of the reach of the SCI PAG resource is incorporated in an evaluation study on the impact of the Canadian Disability Participation Project [29]. Additional resources are needed to monitor the long-term uptake of the resource in different settings across Canada and to evaluate its impact on Canadians with SCI in terms of percentage of Canadians with SCI meeting the starting and advanced levels of the SCI PAG.

### KT challenges

We experienced several challenges in our KT project. The first challenge related to the presentation of the SCI PAG. The panel discussed several ideas to visually/graphically depict the SCI PAG with minimal words, while illustrating how the two guidelines are linked to different benefits and conveying a message like “*Start low, and gradually work your way up*”. Despite working with design experts and generating several prototypes, we were unable to create a simple graphic that clearly conveyed the SCI PAG. We decided, therefore, to present the SCI PAG as a diagram.

The second challenge was balancing between maintaining scientific integrity and creating a clear and useful resource. As described in previous sections, it took us several iterations to develop an evidence-informed diagram of the SCI PAG that was perceived as clear and useful by end-users. To illustrate, our SCI PAG V1 was clearly linked to the scientific evidence, but was not considered as clear and useful. After removing the scientific terms and simplifying the SCI PAG diagram, we were able to create an evidence-informed SCI PAG resource that maintains integrity to the original guidelines but was perceived as clear and useful by a diverse group of potential end-users.

The third challenge was developing an online resource (website). The first author (FH) built the first drafts of the website using a university platform, as this platform was free and relatively easy to work in. As the first author is not a website developer nor a graphic designer, it became challenging to develop an online resource that is accessible, easy to navigate, and interesting to look at. Fortunately, additional funding became available to hire a website developer to improve our online resource prior to the official release of the SCI PAG resource.

### Future directions

Future research is needed to investigate how to develop effective PAG messages targeting the heterogeneous SCI population. Similarly, we need more research on how to implement the SCI PAG with PA promotion and behavior change programs for people with SCI, and how to develop SCI PAG resources that can effectively contribute to increasing PA levels among people with SCI.

We hope that other groups in other countries will translate the SCI exercise guidelines and create supporting resources for their SCI community and share their KT efforts. Subsequently, this may create unique opportunities to compare KT processes between different settings and countries and gain further insights into best KT practices in the context of SCI PAG resources. More specifically, an international comparative study on different SCI PAG resources might contribute to a better understanding of what SCI PAG messages and resources are preferred by different SCI communities in different settings.

## Conclusion

We demonstrated how to use systematic and community-engaged methods to translate the international scientific SCI exercise guidelines into SCI PAG guidelines that are suitable for dissemination and implementation in Canadian community and clinical practice settings. The supporting evidence-informed resource provides user-friendly information on the SCI PAG to help Canadians with SCI achieve the PAG and assist healthcare and fitness professionals, organizations, and policymakers to promote PA among Canadians with SCI. We provide a template for groups in other countries to translate the international scientific SCI exercise guidelines to local settings using a similar systematic and community-engaged approach.

## Supplementary information

Supplementary file

## Data Availability

The quantitative datasets generated and analysed during this study are available in the Open Science Framework (OSF) repository (10.17605/OSF.IO/RYP8E). Summaries of our qualitative data as well as the steps taken to translate the guidelines and develop the supporting resource are published in supplementary files and on OSF.

## References

[1] Martin Ginis KA, Ma JK, Latimer-Cheung AE, Rimmer JH (2016). A systematic review of review articles addressing factors related to physical activity participation among children and adults with physical disabilities. Health Psychol Rev.

[2] Wolfe DL, Martin Ginis KA, Latimer AE, Foulon BL, Eng JJ, Hicks AL. Physical activity and SCI. In: Eng JJ, Teasell RW, Miller WC, Wolfe DL, A.F T, Hsieh JTC, editors. Spinal cord injury, Rehabilitation evidence. Version 3.0. 2010.

[3] Fekete C, Rauch A (2012). Correlates and determinants of physical activity in persons with spinal cord injury: a review using the International Classification of Functioning, Disability and Health as reference framework. Disabil Health J..

[4] Goosey-Tolfrey VL, van der Scheer JW, Lexell J, Clements K, Martin Ginis KA, International SCI Exercise Guidelines Project Group. (2018). Development of scientific exercise guidelines for adults with spinal cord injury. Br J Sports Med..

[5] Martin Ginis KA, van der Scheer JW, Latimer-Cheung AE, Barrow A, Bourne C, Carruthers P (2018). Evidence-based scientific exercise guidelines for adults with spinal cord injury: an update and a new guideline. Spinal Cord..

[6] Ginis KA, Hicks AL, Latimer AE, Warburton DE, Bourne C, Ditor DS (2011). The development of evidence-informed physical activity guidelines for adults with spinal cord injury. Spinal Cord..

[7] Brawley LR, Latimer AE (2007). Physical activity guidelines for Canadians: strategies for dissemination of the message, expectations for change and evaluation. Appl Physiol Nutr Metab.

[8] Latimer AE, Brawley LR, Bassett RL (2010). A systematic review of three approaches for constructing physical activity messages: What messages work and what improvements are needed?. Int J Behav Nutr Phys Act..

[9] Gagliardi AR, Brouwers MC, Bhattacharyya OK (2014). Guideline implementation R, application N. A framework of the desirable features of guideline implementation tools (GItools): Delphi survey and assessment of GItools. Implement Sci..

[10] Harrison MB, Legare F, Graham ID, Fervers B (2010). Adapting clinical practice guidelines to local context and assessing barriers to their use. CMAJ.

[11] Leone L, Pesce C. From delivery to adoption of physical activity guidelines: realist synthesis. Int J Environ Res Public Health. 2017;14:1–19. 10.3390/ijerph14101193 PMC5664694 28991184

[12] Straus SE, Tetroe J, Graham ID. Knowledge translation in health care. Moving from evidence to practice. West Sussex, UK: John Wiley & Sons; 2013.

[13] Brouwers MC, Kho ME, Browman GP, Burgers JS, Cluzeau F, Feder G (2010). AGREE II: advancing guideline development, reporting and evaluation in health care. CMAJ.

[14] Arbour-Nicitopoulos KP, Martin Ginis KA, Latimer-Cheung AE, Bourne C, Campbell D, Cappe S (2013). Development of an evidence-informed leisure time physical activity resource for adults with spinal cord injury: the SCI Get Fit Toolkit. Spinal Cord..

[15] Ginis KA, Heisz J, Spence JC, Clark IB, Antflick J, Ardern CI (2017). Formulation of evidence-based messages to promote the use of physical activity to prevent and manage Alzheimer’s disease. BMC Public Health.

[16] Latimer-Cheung AE, Rhodes RE, Kho ME, Tomasone JR, Gainforth HL, Kowalski K (2013). Evidence-informed recommendations for constructing and disseminating messages supplementing the new Canadian Physical Activity Guidelines. BMC Public Health.

[17] Brouwers MC, Kerkvliet K, Spithoff K, Consortium ANS (2016). The AGREE reporting checklist: a tool to improve reporting of clinical practice guidelines. BMJ.

[18] Martin Ginis KA, Latimer AE, Arbour-Nicitopoulos KP, Buchholz AC, Bray SR, Craven BC et al. Leisure time physical activity in a population-based sample of people with spinal cord injury part I: demographic and injury-related correlates. Arch Phys Med Rehabil. 2010;91:722–8. 10.1016/j.apmr.2009.12.027 20434609

[19] Martin Ginis KA, Arbour-Nicitopoulos KP, Latimer AE, Buchholz AC, Bray SR, Craven BC et al. Leisure time physical activity in a population-based sample of people with spinal cord injury part II: activity types, intensities, and durations. Arch Phys Med Rehabil. 2010;91:729–33. 10.1016/j.apmr.2009.12.028 20434610

[20] Best KL, Arbour-Nicitopoulos KP, Sweet SN. Community-based physical activity and wheelchair mobility programs for individuals with spinal cord injury in Canada: Current reflections and future directions. J Spinal Cord Med. 2017;40:777–82. 10.1080/10790268.2017.1367363 PMC5778941 28872428

[21] van der Scheer JW, Martin Ginis KA, Ditor DS, Goosey-Tolfrey VL, Hicks AL, West CR et al. Effects of exercise on fitness and health of adults with spinal cord injury: A systematic review. Neurology. 2017;89:736–45. 10.1212/WNL.0000000000004224 28733344

[22] Foulon BL, Lemay V, Ainsworth V, Martin Ginis KA (2012). Enhancing physical activity guidelines: a needs survey of adults with spinal cord injury and health care professionals. Adapt Phys Act Q..

[23] Clegg D, Barker R. Case Method Fast-Track: A RAD Approach, Addison-Wesley, Boston, MA, United States, 1994.

[24] Hoekstra F, Ginis KM. Physical activity guidelines for adults with spinal cord injury. OSF. 2019. 10.17605/OSF.IO/RYP8E.

[25] Gagliardi AR, Marshall C, Huckson S, James R, Moore V (2015). Developing a checklist for guideline implementation planning: review and synthesis of guideline development and implementation advice. Implement Sci..

[26] Escoffery C, Lebow-Skelley E, Udelson H, Boing EA, Wood R, Fernandez ME (2019). A scoping study of frameworks for adapting public health evidence-based interventions. Transl Behav Med.

[27] Moore AE, Straus SE, Kasperavicius D, Bell NR, Dickinson JA, Grad R (2017). Knowledge translation tools in preventive health care. Can Fam Phys.

[28] Brouwers M, Stacey D, O’Connor A (2010). Knowledge creation: synthesis, tools and products. CMAJ.

[29] Hoekstra F, Martin Ginis KA, Allan V, Kothari A, Gainforth HL (2018). Evaluating the impact of a network of research partnerships: a longitudinal multiple case study protocol. Health Res Policy Syst..

[30] Camden C, Shikako-Thomas K, Nguyen T, Graham E, Thomas A, Sprung J, et al. Engaging stakeholders in rehabilitation research: a scoping review of strategies used in partnerships and evaluation of impacts. Disabil Rehabil. 2015;37:1390–400. 10.3109/09638288.2014.963705. 10.3109/09638288.2014.963705 25243763

