# Supplementary material for: Translating the international scientific spinal cord injury exercise guidelines into community and clinical practice guidelines: a Canadian evidence-informed resource
Source: Spinal Cord. 2020 Jan 16;58(6):647–57. doi: 10.1038/s41393-019-0410-1 (PMC7283041; doi:10.1038/s41393-019-0410-1)
Supplement: Supplementary file 1 — Supplementary file [file 41393_2019_410_MOESM1_ESM.pdf]

Supplementary files: The Canadian SCI Physical Activity Guidelines

List of appendices

- **Appendix 1**: The application of the AGREE II items to translate the scientific guidelines to community and clinical practice guidelines
- **Appendix 2:** The AGREE reporting checklist
- **Appendix 3**: Expert panel members' names, organizations, expertise and roles.
- **Appendix 4**: Criteria for selecting the evidence-base
- **Appendix 5:** Agenda of the one-day consensus meeting
- **Appendix 6:** Summarizing key recommendations of the online resource and supporting evidence
- **Appendix 7**: Link between content of the online resource and supporting evidence
- **Appendix 8:** Procedures for pilot tests
- **Appendix 9:** Results from survey with panel members (n=6) – Pilot 1
- **Appendix 10:** Summary of panel members' and participants' feedback on the first draft of the guidelines (SCI PAG V1) – Pilot 1
- **Appendix 11**: Summary of panel members' feedback on the second draft of the guidelines (SCI PAG V2) – Pilot 2
- **Appendix 12:** Results from interviews with individuals with SCI (n=8) – Pilot 3
- **Appendix 13:** The translation of the international scientific SCI exercise guidelines to community and clinician practice guidelines

Supplementary files: The Canadian SCI Physical Activity Guidelines

Appendix 1: The application of the AGREE II items to translate the scientific guidelines to community and clinical practice guidelines.

AGREE II item*	Revised AGREE II item*	Application to the updated physical activity guidelines resource
Domain 1: Scope and Purpose		
1. The overall objective(s) of the guideline is (are) specifically described.	The overall objectives of the message recommendations are specifically described.	To provide an evidence-informed resource that includes user-friendly information on the scientific SCI exercise guidelines and explains how adults with SCI can implement the exercise guidelines into their lifestyle.
2. The health question(s) covered by the guideline is (are) specifically described.	The practical question(s) covered by the message recommendations is (are) specifically described.	1) What type of information is needed to support the dissemination and implementation of the SCI exercise guidelines in Canadian community and clinical settings?<br>2) How should this information be formatted and delivered to adults with SCI living in Canada?
3. The population (patients, public, etc.) to whom the guideline is meant to apply is specifically described.	The population to whom the message recommendations are targeted is specifically described.	Adults with SCI (all levels of injury up to C3) who are 18 to 64 years old, and have been injured more than one year, including adults with SCI who are not yet meeting the PAG. Of note, the target population mirrors the target population of the scientific SCI exercise guidelines [1].
Domain 2: Stakeholder Involvement		
4. The guideline development group includes individuals from all relevant professional groups.	The message recommendations development group includes individuals from all relevant professional groups.	An expert panel included of adults with SCI (AIS A-D); experts in exercise, PA, behaviour change, physiology, and KT; clinician; representatives from SCI organizations, and academic researchers assisted in this project (**Appendix 3**).
5. The views and preferences of the target population (patients, public, etc.) have been sought.	The views and preferences of the target population (i.e., end-users) have been sought.	This KT project was guided by findings from previous stakeholder-engaged projects related to the development and dissemination of SCI PAG [1-4].<br>A diverse expert panel assisted in the KT of the scientific SCI exercise guidelines. Our KT project included a variety of pilot tests with adults with SCI and other end-users (**Figure 1**). Ultimately, stakeholder engagement processes resulted in feedback from nearly 100 adults with SCI and other end-users (**Tables 1, 2, and appendix 12**).
6. The target users of the guideline are clearly defined.	The target users of the messages are clearly defined.	Adults with SCI, family members, friends and caregivers of adults with SCI, clinicians, practitioners, rehabilitation centres, community organizations, federal and provincial sport and health ministries, Canadian Parks and Recreation Association, Canadian Society for Exercise Physiology,

		Young Men's Christian Association (YMCA) and other public and private fitness centers.
Domain 3: Rigour of Development		
7. Systematic methods were used to search for evidence.	Systematic methods were used to search for evidence.	The articles describing the development of the scientific SCI exercise guidelines [1, 5, 6] provided the starting point to review the evidence-base. A purposive search strategy was conducted to review the evidence-base focusing on the following three topics:<br>1) Development and/or dissemination of the SCI PAG<br>2) PA participation, benefits, and determinants relevant for Canadians with SCI<br>3) Development and adaptation of PAG messages.
8. The criteria for selecting the evidence are clearly described.	The criteria for selecting evidence are clearly described.	Criteria for selecting evidence-base are outlined in **Appendix 4.**
9. The strengths and limitations of the body of evidence are clearly described.	The strengths and limitations of the body of evidence are clearly described.	Strengths include:<br>1) the use of community-engaged methods to develop the previous and updated PAG [1, 2, 4]<br>2) Canadian-specific findings on PA determinants and preferences [7, 8], an overview of evidence-informed PA programs for people with SCI [9], and experimental evidence supporting the use of SCI-specific behaviour change programs.<br>3) Availability of systematic reviews on what should be included in effective PAG messaging [10, 11].<br>Limitations include:<br>1) Insufficient evidence to support fitness or health benefits of physical activity for acute SCI [1, 6]<br>2) Insufficient evidence to draft recommendations specific for different types of activities [1, 6]<br>3) A lack of SCI-specific systematic reviews on effective PAG messaging.
10. The methods for formulating the recommendations are clearly described.	The methods for formulating the recommendations are clearly described.	The methods included the following steps:<br>• Panel members reviewed key evidence prior to the one-day meeting.<br>• Project lead provided a presentation about the development of the scientific exercise guidelines at the beginning of the consensus meeting.<br>• Panel members discussed information that potentially could be included in the resource in small working groups.

		• Project lead provided a summarizing overview of key decisions and panel members reviewed and revised those.<br>• Three panel members (KMG, FH, SG) held a debriefing meeting one week after the one-day meeting to further refine the information and prepare the next steps of the development process.
11. The health benefits, side effects, and risks have been considered in formulating the recommendations.	The direct practical implications have been considered in formulating the recommendations.	The practical implications that are considered are:<br>1) Additional support and resources might be needed to promote advanced level of the PAG (3x/week). The advanced level might overwhelm adults with SCI who are not yet doing any PA.<br>2) Additional support and resource to hire a technical writer, graphic designer and website developer to help develop the SCI PAG resource.<br>3) Additional support and resources needed to train and support healthcare and fitness professionals.
12. There is an explicit link between the recommendations and the supporting evidence.	There is an explicit link between the messaging recommendations and the supporting evidence.	**Appendices 6 and 7** describe the link between recommendations in the SCI PA guidelines resource and the supporting evidence.
13. The guideline has been externally reviewed by experts prior to its publication.	The message recommendations have been externally reviewed by experts prior to its publication.	The complete SCI PAG resource has been reviewed by expert panel members and individuals with SCI (n=8). Revisions were applied accordingly. The information related to the SCI PAG (Layer 1) was reviewed by a diverse group of potential end-users (n=90). The results are presented in **Tables 1, 2, and appendix 12**.
14. A procedure for updating the guideline is provided.	A procedure for updating the message recommendations is provided.	The SCI PAG and supporting resource will be updated at the same time as the scientific SCI exercise guidelines. The hyperlinks to other existing resources will be updated once a year.
Domain 4: Clarity of Presentation		
15. The recommendations are specific and unambiguous.	The recommendations are specific and unambiguous.	The recommendations provided in the resource are considered as clear by panel members, adults with SCI, and other potential end-users (see **Tables 1, 2, and appendix 12**)
16. The different options for management of the condition or health issue are clearly presented.	The different options for developing and disseminating messages are clearly presented.	The panel recommended the development of an online resource in which the information is presented in "layers" in order to tailor the information to the needs and preferences of end-users (see **Figure 2**).<br>The panel also suggested the development of a paper version in postcard format including the key PAG recommendations

		(layer 1 information). Based on the results of pilot 2 and 4, the development of such a paper version did not yet get our priority. We did create PDF documents in English and French of the SCI PAG and the FAQs.
17. Key recommendations are easily identifiable.	Key recommendations are easily identifiable.	The Home Page of the online resource presents the diagram with the SCI PAG (www.sciguidelines.com or www.sciguidelines.ca)
Domain 5: Applicability		
18. The guideline describes facilitators and barriers to its application.	Facilitators and barriers to the application of the recommendations were discussed.	Facilitators and barriers are discussed with the expert panel during the one-day meeting and outlined in the discussion section.
19. The guideline provides advice and/or tools on how the recommendations can be put into practice.	The message recommendations provide advice and/or tools on how the recommendations can be put into practice.	The SCI PAG resource is free available via www.sciguidelines.com or www.sciguidelines.ca and can be accessed via computer, table, or mobile-device. The resource includes specific menu for people with SCI and for healthcare professionals.
20. The potential resource implications of applying the recommendations have been considered.	The potential resource implications of applying the recommendations have been considered.	The panel discussed the resources needed to disseminate the updated SCI PAG and to develop related resources during the consensus meeting. The potential resources are described in the **discussion section**.
21. The guideline presents monitoring and/or auditing criteria.	Strategies for monitoring and/or auditing the uptake of the recommendations was considered by the expert panel.	The panel discussed the monitoring and evaluation of the uptake of the SCI PAG resource during the consensus meeting. The use of the SCI PAG will be assessed by monitoring the number of views of the website as well as the number of downloads of the PDF-versions of the guidelines.<br>The use and uptake of the SCI PA guidelines will be evaluated if financial resources are available.
Domain 6: Editorial Independence		
22. The views of the funding body have not influenced the content of the guideline.	The views of the funding body have not influenced the content of the message recommendations.	The consensus panel did not include representatives from the funding agencies.
23. Competing interests of guideline development group members have been recorded and addressed.	Competing interests of message recommendation development group members have been recorded and addressed.	Panel members did not report any conflict of interests.

Notes: PA = Physical Activity, PAG = Physical Activity Guidelines, SCI = Spinal Cord Injury. *Definitions are derived from the original AGREE II [12] and the revised AGREE II [13]

Supplementary files: The Canadian SCI Physical Activity Guidelines

Appendix 2: The AGREE II Reporting Checklist.

The AGREE II Reporting Checklist is included at the end of this document.

Appendix 3: Expert panel members' names, organizations, expertise and roles.

Name	Organization(s)	Expertise	Role(s)
Kathleen Martin Ginis, PhD	ICORD, University of British Columbia	Researcher with expertise in SCI, PA Behaviour change, KT	Content expert, project lead, chair, writing team
Femke Hoekstra, PhD	ICORD, University of British Columbia	Researcher with expertise in SCI, PA promotion, KT	Content expert, writing team, resource development, pilot testing
Jaimie Borisoff, PhD	ICORD, University of British Columbia	SCI end-user (manual wheelchair user) and researcher with expertise in	Content expert, feedback team
Mary Jo Fetterly	-	SCI end-user (power and manual wheelchair user)	Content expert, feedback team
Spero Ginis	University of British Columbia	KT Specialist	Content expert, photographer, feedback team
Amy Latimer-Cheung, PhD*	Queen's University	Researcher with expertise in SCI, PA guideline development, messaging and behaviour change	Content expert, feedback team
Jasmin Ma, PhD	University of British Columbia	Researcher with expertise in SCI, PA Behaviour change, KT	Content expert, feedback team
Jocelyn Maffin	SCI BC	SCI end-user (manual wheelchair user)	Content expert, feedback team
Lorne Mah*	-	SCI end-user (ambulatory SCI)	Content expert, feedback team
Christopher B McBride, PhD	SCI Canada, SCI BC	Executive director of SCI community organization	Project lead, chair, feedback team
Christopher West, PhD	ICORD, University of British Columbia	Researcher with expertise in SCI, exercise, cardiovascular health; exercise rehabilitation	Content expert, feedback team
Rhonda Willms, PhD	ICORD, GF Strong, University of British Columbia Medicine	Clinician and researcher	Content expert, feedback team

Notes: PA = Physical Activity, SCI = Spinal Cord Injury; ICORD = International Collaboration on Repair Discoveries. KT = Knowledge Translation. *Amy Latimer-Cheung attended parts of the consensus meeting via conference call. Lorne Mah did not attend the consensus meeting, but participated in the focus group discussion.

Appendix 4: Criteria for selecting and reviewing the evidence-base

Topic	Selection criteria
1) Development and/or dissemination of SCI PAG	- SCI-specific papers focusing on the development and/or dissemination of the first SCI fitness guideline or focusing on the scientific SCI exercise guidelines.
2) PA participation and promotion, PA benefits, PA determinants and preferences	- SCI-specific articles used to develop the previous PAG resource for adults with SCI in Canada<br>*OR*<br>- SCI-specific systematic reviews focusing on PA participation, PA promotion, PA benefits, PA determinants and/or PA preferences<br>*OR*<br>- SCI-specific articles conducted in Canadian context focusing on PA participation, PA promotion, PA benefits, PA determinants, and/or PA preferences.
3) Development and adaptation of PAG messaging	- Systematic reviews focusing on the development, adaptation, and/or delivery or PAG messaging.

PA = Physical Activity; SCI = Spinal Cord Injury; PAG = Physical Activity Guidelines.

Appendix 5: Agenda of the one-day consensus meeting (April 23, 2018 – Vancouver)

**Time**	**Item**
09:30-09:45	**1. Welcome, Introductions, Meeting Objectives, Conflict of Interest**
09:45-10:15	**2. Background and Methods**<br>- SCI Get Fit Toolkit Origins<br>- What's 'new' in the new SCI Exercise Guidelines<br>- Consumer preferences for guideline-supporting resources<br>- The AGREE-II process
10:15-11:15	**3. Breakout session #1: Discussion of Existing Resource Content** (2 Groups)<br>Guidelines** Activity ideas Benefits of Physical Activity<br>Action Planning Safety Tips Overcoming Barriers
11:15-11:30	**4. Break**
11:30-12:30	**5. Groups Reconvene to Discuss Results of Breakout Session #1**
12:30-1:00	**6. Lunch**
1:00-1:45	**7. Breakout session #2: Discussion of Potential New Content**<br>Sample programs Instructional videos<br>Links to Resources Other
1:45-2:30	**8. Groups Reconvene to Discuss Results of Breakout Session #2**
2:30-2:45	**9. Break**
2:45-3:30	**10. General Recommendations for Resources**<br>Target Audiences Resource format, type, length<br>Key messages Images
3:30-4:15	**11. Practical applicability (AGREE items)**<br>- How resource(s) can be disseminated/put into practice.<br>- Facilitators and barriers to application.<br>- Potential resource implications.<br>- Monitoring and/ or auditing criteria.
4:15-5:00	**12. Moving forward (AGREE items)**<br>- Plans for updating<br>- Review by stakeholders and experts<br>- Missing expertise
	**Next steps**

Notes: Other information related to the consensus meeting is available via Open Science Framework [14]

Appendix 6: Summarizing key recommendations on the online resource and supporting evidence

Topic	Summarizing key recommendations	Supporting evidence*
**Target audiences**	Information should primarily focus on adults with SCI (all levels of injury up to C3) who are 18 to 64 years old, and have been injured more than one year, including adults with SCI who are not yet meeting the PAG.	[1, 5, 6]
**Educational information**	The information should:<br>- Use simple language to clarify how much PA adults with SCI should be doing (i.e. the SCI PA guidelines)<br>- Clarify key terms used in the guidelines (e.g., moderate and vigorous intensity, aerobic and strength training)	[1, 3, 5]
**Motivational information**	The information should:<br>- Reflect the overarching message “Start slow and gradually increase the amount, intensity, and/or frequency of PA”	[1, 5]
	- Highlight the benefits of PA, with a particular emphasize on benefits on daily activities (e.g., easier transfers)	[6, 15-17]
	- Provide adults with SCI a variety of ways to meet the SCI PAG	[7, 8, 18, 19]
	- Provide a variety of PA examples for ambulatory SCI, manual wheelchair users, and power wheelchair users including aerobic activities and strength-training activities, home-based activities, in- and outdoor activities, and sports	[7, 8, 18, 19]
	- Provide clear tips and resources on how to become more active, including action planning and goal setting	[9, 20-22]
	- Use empowering messages and should be all to action (e.g., “You can do this”, “It’s easier than you think”)	[10, 13]
	- Highlight ways to overcome barriers that people with SCI may perceive	[3, 20, 21]
	- Provide information on how to participate in PA safely	[3, 23]
	- Provide instructions on how and when to contact SCI community organizations	
**Information delivery**	Information should:<br>- Be delivered in a tailored way based on persons’ needs and preferences (e.g., people who are already meeting the guidelines vs people who are not yet meeting the guidelines)<br>- Be delivered via an online mobile-friendly website in which information is presented in “layers”<br>- Deliver information on how many PA individuals should be doing in printed postcard format<br>- Include links to existing evidence-informed resources, such as video’s and manuals (“*Do not re-invent the wheel*”)	[3, 24]
**Lay-out**	The resource should:<br>- Include a variety of images	[3]

	- Use simple language	[3]
	- Limited amount of text (more graphics than words)	[3]
	- Include quotes from peers (testimonial style) who are physically active	[25]

Note: PA = Physical Activity; SCI = Spinal Cord Injury. * Only key references are presented. **Appendix 7** includes additional information on the link between the information in the resource and the supporting evidence.

Appendix 7: Link between content of the online resource and supporting evidence

Topic	Content	Supporting evidence
**Physical activity guidelines**	Physical activity guidelines:<br>- Beginner level reflects the fitness guidelines<br>- Advanced level reflects the combined fitness and cardiometabolic health guidelines	[1, 5, 6]
**Physical activity benefits**	Physical activity benefits on daily activities and general health benefits.	Reviews:<br>[6, 15-17]<br>Other papers:<br>[3, 23, 26]
**Activity examples**	- Activity examples are grouped into every day activities, accessibility activities, and adapted sports<br>- Examples are provided for individuals with ambulatory SCI, manual wheelchair users, and power wheelchair users.	[7, 8, 18, 19]
**Overcoming barriers**	Common barriers identified:<br>- Lack of time<br>- Lack of motivation<br>- Physical barriers<br>- Lack of accessible facilities<br>- Lack of support and/or knowledge	Reviews:<br>[20, 21]<br>Other papers:<br>[25, 27-32]<br>[27-32]
**Tools to success** *(strategies to promote physical activity)*	Key tools:<br>- Goal setting and action planning<br>- Contact community organizations, rehabilitation centre and/or peers for support<br>- Try different types of physical activities<br>- Be active with friends or family<br>- Physical activity is fun	Reviews:<br>[9, 20-22]<br>Other papers:<br>[25, 27-32]
**Safety tips**	Safety tips should highlight:<br>- Risk of overuse injury<br>- Information on proper technique<br>- Check skin<br>- Hydration<br>- Specific safety tips for individuals with SCI at or above T6	[3, 23]

Appendix 8: Pilot tests procedures

The clarity of the presentation, usability and appropriateness of the spinal cord injury (SCI) physical activity guidelines (PAG) and supporting resource were pilot tested at various stages of development with different groups of potential end-users. This appendix outlines the procedures of the different pilot tests.

Pilot test 1: Clarity and appropriateness of the SCI PAG version 1 (V1)

Procedures: After drafting the first version (V1) of the SCI PAG (July 2018), panel members and adults with SCI were invited to review the SCI PAG V1. Panel members completed an online survey including questions related to the clarity of the presentation of the SCI PAG V1. The survey items are available via **OSF**. In addition to this online survey, we invited adults with SCI to review the SCI PAG V1 and provide feedback via a telephone-based interview with the first author (FH). After receiving feedback from six panel members and three adults with SCI (all manual wheelchair users), the results showed that the survey questions related to the clarity of the guidelines were “mixed” (see **Appendix 9** and **OSF**) and that a substantial revision of the SCI PAG was needed to address participants’ feedback. Therefore, we did not invite more people to review SCI PAG V1, but decided to focus first on drafting a new version.

Pilot 2: Clarity, usability and appropriateness of SCI PAG version 2 (V2) and supporting resource

Procedures: After drafting the second version (V2) of the SCI PAG and a first version of the content of the supporting resource (January 2019), panel members were invited to

review the SCI PAG V2 and supporting resource. Panel members (n=11) completed an online survey including questions related to the clarity, usability and feasibility of the SCI PAG V2 and supporting resource. The survey items are available via OSF.

Pilot 3: Clarity, usability, and appropriateness of SCI PAG version 2 (V2) and supporting resource

<u>Procedures:</u> A diverse group of adults with SCI was invited to review the SCI PAG V2 and supporting resource via an in-person interview conducted by the first author (FH) Participants were asked to navigate through the online resource and ‘think’ aloud. In addition, the first author (FH) asked specific questions related to the clarity, usability, and appropriateness of the SCI PAG V2 and supporting resource. Interviews were audio-recorded. Participants’ feedback was summarized using the notes made by first author (FH) and by listening to the recordings of the interviews. After eight interviews we noticed that all participants were generally positive about the resource and provided similar directions to improve the resource (e.g., navigation, lay-out – see **appendix 12**). Therefore, we decided to address their feedback and move forward to the next pilot test after interviews conducting 8 interviews. Participants received a $10 gift card for completing the interview.

<u>Participants:</u> Eights healthy adults (5 males and 3 females) with chronic SCI participated in the pilot test. Participants’ primary mode of mobility was: manual wheelchair (n=6), power wheelchair user (n=1), and independent walker (n=1). All participants engaged in different types of physical activities and/or sports. Although we did not interview adults who did not engage in any type of physical activities, participants mentioned that they

know other people with SCI who are not yet doing any physical activity and that they would share the resource with them.

Pilot 4: Clarity, usability and appropriateness of the SCI PAG V2

Procedures: A diverse group of potential end-users was invited to review the SCI PAG V2 and complete a short online survey focusing on the clarity, usability and appropriateness of the SCI PAG V2 and inspired by survey questions used in previous SCI PAG surveys as well as guided by the AGREE II instrument. Participants received a $5 gift card for completing the survey. For financial reasons, we aimed to recruit a maximum of 100 participants.

Participants: 90 potential end-users completed the survey. Participants' demographics are presented in **Table 2**.

Analyses of survey and interview data

Responses to survey items are reported using medians and interquartile range (IQR). Summaries of panel members' and participants' qualitative feedback including information on how we addressed the feedback are presented in **Tables 1 and 2** as well as in our supplementary files (**Appendices** and **OSF**).

Appendix 9: Results from survey with panel members (n=6) on the first draft of the guidelines (V1) – Pilot 1

Questions	Median (IQR)	Range
General questions		
- Is the resource appropriate for adults with SCI who are not doing any physical activity?	5.0 (2.0)	3-7
- Is the resource appropriate for adults with SCI who are doing some physical activity?	6.0 (1.0)	5-7
- Does the resource provide simple messages about physical activity?	5.5 (1.0)	5-6
- Does the resource provide clear information on what to do if an adult with SCI wants to improve his/her fitness and/or cardiometabolic health?	5.5 (1.0)	3-6
Fitness guidelines		
- Does the resource provide clear instructions about how much physical activity should be done in a week to improve fitness?	6.5 (2.0)	4-7
- Does the resource provide clear instructions about the intensity level of physical activity to improve fitness?	5.5 (1.0)	5-7
- Does the resource provide clear instructions about how much physical activity should be done in one session to improve fitness?	6.5 (2.0)	2-7
Cardiometabolic health guidelines		
- Does the resource provide clear instructions about how much physical activity should be done in a week to improve cardiometabolic health?	7.0 (1.0)	6-7
- Does the resource provide clear instructions about the intensity level of physical activity to improve cardiometabolic health? (2)	5.5 (1.0)	5-7
- Does the resource provide clear instructions about how much physical activity should be done in one session to improve cardiometabolic health?	6.0 (2.0)	2-7
Image		
- Does the image (the blue diagram) provide a clear summary of the Fitness Guidelines and the Cardiometabolic Health Guidelines?	6.0 (2.0)	5-7
- Does the image provide clear instructions on how the Fitness Guidelines and Cardiometabolic Health Guidelines can be combined?	4.5 (4.0)	3-7
- Does the image provide appropriate information for adults with SCI who are not doing any physical activity?	4.5 (2.0)	3-6
Logo		
- Does the resource include an appropriate logo (i.e. the logo at the top of the document)?	5.0 (2.0)	4-6
Questions and answers (FAQ)		
- Does the resource use language that is appropriate for adults with SCI who are not doing any physical activity?	6.0 (2.0)	5-6

- Does the resource use language that is appropriate for adults with SCI who <u>are</u> doing some physical activity?	6.0 (1.0)	6-7
- Does the FAQ include appropriate questions and answers for adults with SCI who <u>are not</u> doing any physical activity?	5.5 (1.0)	5-7
- Does the FAQ include appropriate questions and answers for adults with SCI who <u>are</u> doing some physical activity?	6.0 (1.0)	6-7

Notes: Survey questions are answered on a 7-point Likert scale, in which a higher response indicates a more positive response (e.g., 1 = strongly disagree, 4 = neither agree, nor disagree, 7 = strongly agree). The physical activity guidelines version 1 (V1) included the fitness guideline, the cardiometabolic health guideline, and a combined guideline (see **OSF**).

Appendix 10: Summary of panel members' and participants' feedback on the first draft of the guidelines (SCI PAG V1) – Pilot 1

Topic	Panel members' feedback	Response to feedback
Guidelines	- The information is not really focused on adults with SCI who are not yet doing any physical activity	We included specific information for adults with SCI who are not yet doing any physical activity.
	- The combined guidelines are not clear. It looks like that adults with SCI are required to be active 5 days/week to meet the combined guidelines.	We changed diagram and removed the combined guidelines (see SCI PAG V2)
	- It is not clear what the benefits are	Details on physical activity benefits are presented on a separate page and not included in Pilot 1.
	- Intensity definitions are not clear	
	- Intensity definitions should be presented at the first page	
	- Add details on injury level and completeness of injury in the FAQ section.	
	- Add additional information on what should be done in one session	An example of an action plan is included on the website.
	- There might be value in sharing a graphic representation of why it's important to participate in the guidelines	Testimonials and images are included throughout the website.
	- The rational for bout must be at least 5 min in duration is not clear	We removed the 5 minutes duration.
Image	- Image does not contain information tailored to adults with SCI who are not doing any physical activity.	
	- Be clear that the guidelines are minimum levels of activity needed to achieve benefits	
Lay out	- The lay out looks like a scientific report.	
	- The length of the resource (2 pages) will limit it use to certain delivery opportunities.	We have separated the guidelines from the FAQs and presented the information on two separate pages.
Logo	- Logo is not inclusive	We revised the logo and included ambulatory SCI.
	- Canadian flag in the logo could be removed	We did not remove the Canadian flag as it illustrates that the guidelines are specifically developed for adults with SCI living in Canada.

Notes:* If no response is presented, revisions are made accordingly. The physical activity guidelines version 1 (V1) included the fitness guideline, the cardiometabolic health guideline, and a combined guideline (see **OSF).

Appendix 11: Summary of panel members' feedback on the second draft of the guidelines (SCI PAG V2) – Pilot 2

Website page	Suggested improvements	Response*
	**Content**	
Physical Activity Guidelines (Home page)	- It is not clear that both guidelines (starting and advanced) reflect a minimum for different goals (Fitness and Cardiometabolic Health)	The minimum levels are only highlighted for the 'starting' level because this level reflects the Fitness Guidelines. The 'advanced' level reflects the combined guidelines (Fitness and Cardiometabolic Health Guidelines), and not the Cardiometabolic Health Guidelines alone.
	- There is not so much information on pre-intenders	We moved information for adults who are not yet doing any physical activity to the top of the page.
	- The word 'reps' in the diagram might not be clear for general population	We clarified this in the FAQs.
Physical Activity Guidelines Explained (FAQs)	- The word 'additional' benefits implies a dose response relationship, but there is no evidence for a dose response relationship	In order to keep the guidelines as simple as possible, we did not change this.
	- Include information on how to combine aerobic and strength training	Examples are provided on the action planning page and activity examples.
Benefits of Physical Activity	- With respect to the evidence, it would be nice to have brief lay summaries of most salient papers	We included a link to an existing resource providing summarizing evidence on benefits.
Activity Examples	- Include a separate page for power wheelchair users and acknowledge some of the differences and challenges.	We included a separate page for power wheelchair users.
	- The activity ramp suggests that you should work up to sports.	We removed the numbers on the ramp to focus on more on the three categories instead of working up to the sports category.
	- Practise Yoga, Pilates or Tai Chi are not strength training exercise, but flexibility exercises	
Your keys to success	- Include instructions on the template for action planning.	
Overcoming barriers	- Sharing peer stories, experiences, or examples could add further value.	We included a testimonial on this page.
Safety tips	- Why is there only information on AD and not on exercise induced hypotension for injuries about T6.	As we did not want to include too much information on safety tips, we only provided limited information on this page and included links to additional resource with information on exercise induced hypotension and other safety tips. We changed the order of the safety tips and highlighted the most important information.
Resources	- Encourage people to contact their local recreation centre guide	

	Navigation and lay-out		
Navigation	-	Improve navigation – too many clicks.	We changed the menu in order to improve the navigation.
	-	Include images in website without a click	
Logo	-	The power wheelchair users are not represented in the logo	We changed the logo.
	-	The individual with ambulatory SCI is in the background which implies that this group is less important.	
Lay-out	-	The website looks old-fashioned.	We hired a website developer to update the website.
Testimonials	-	Include more testimonials	

**Note*: If no response is presented, revisions were made accordingly.

Appendix 12: Results from interviews with individuals with SCI (n=8) – Pilot 3

Interview survey-items	Agreement	Suggested improvements	Response***
General objectives			
- Is the resource appropriate for adults with SCI who are not yet doing any physical activity?	8/8 (100%)		
- Is the resource appropriate for adults with SCI who are doing some physical activity?	8/8 (100%)		
- Does the resource reflect the overarching message “Start slow and gradually increase the amount of physical activity”?	7/8 (88%)		
- Does the resource teach adult with SCI how to make smart and informed choices about being physically active?	8/8 (100%)		
- Does the resource encourage adults with SCI how to make smart and informed choices about being physically active?	8/8 (100%)		
- Does the resource provide adults with SCI a variety of ways to meet the PA Guidelines?	7/7 (100%)		
Physical Activity Guidelines (Home page)			
- Is it clear how much physical activity individuals with SCI should be doing?	8/8 (100%)	The blue box with 'MEET' is presented between the two levels. It is not clear where the blue box is referring to and when people are meeting the guidelines	We changed this in the final SCI PAG.
- Does the image (the diagram) provide a clear summary of the two levels of the physical activity guidelines?	8/8 (100%)	Add information on ‘how to start if you are not yet doing any physical activity’ on the home page.	We did not include this information on the home page, but we re-organized the menu of the website and included the FAQs in the main menu.
Physical Activity Guidelines (FAQ)			
		Move the box with information “I don’t do any physical activity at all. How should I start?” to the top.	We have changed this accordingly.
		Include the mild – moderate – vigorous intensity terms to the RPE scale.	Additional information is included to the RPE scale.
Logo			
- Does the resource include an appropriate logo?	6/8 (75%)	The power wheelchair users are not included in the logo	The final logo includes all three SCI groups.

Activity examples			
Does the resource include a variety of physical activity examples:			
- For ambulatory SCI?	8/8 (100%)		
- For manual wheelchair users?	8/8 (100%)		
- For power wheelchair users?	3/6 (50%) *	Add separate section with activity examples for tetraplegia / power wheelchair users or add symbols to every activity which are accessible for power and/or manual chairs.	We created a separate page with activity examples for power wheelchair users.
		Include gardening and house holding activities to 'everyday activities' and include more examples of sports	
		Include instructions about how to do strengthening and aerobic activities at home	We created a separate page with links to the Active Home guides and videos.
Benefits, overcoming barriers, safety tips			
- Are the benefits identified of value for you?	8/8 (100%)	Add information on benefits on mental health	We included statement "Improved mental health and lower risk of depression"
		Add information on benefits on gut/ bladder/ bowel function	We did not include this information as there is limited scientific evidence to support this statement.
- Does this page [overcoming barriers] include useful and valuable information?	8/8 (100%)	Add a testimonial to the barrier-page	We included more testimonials.
- Does this page [safety tips] include useful and valuable information?	6/6 (100%)	Include information on warming-up and cooling-down	We added this statement and included a link to examples of warming-up exercises.
Keys to success (action planning)			
		Reduce the amount of the text on 'Action Planning' page. The page suggests that Action Planning is very complicated.	We reduced the text on the website and included additional information in a separate document which includes the template.
Resources			
		List resources on alphabetical order	
		Include regional information about exercise facilities for people with SCI	It was not feasible to include this information for all regions in Canada. But links to contact information of provincial SCI organizations are included throughout the website.
Navigation			

		Include links to contact information of provincial SCI organizations throughout the website	We added a box with contact information on different pages of the website.
		Include links to 'more information' when clicking on the diagram and/or images.	When clicking on the diagram, a PDF-version of the SCI PAG will be downloaded.
Presentation, language			
- Is the information in the resource presented in an appropriate and encouraging way?	8/8 (100%)	Use a larger font size throughout the website	We worked together with a website developer was to improve the fonts and layout.
		Include a banner with photos on the home page	The website developer created a banner on the homepage.
		Present images as a collage	
Usability			
- Will you use this resource?	8/8 (100%)		

Note: Participants' primary mode of mobility was: manual wheelchair (n=6), power wheelchair user (n=1), and independent walker (n=1). This variation in mode of mobility illustrates our diverse sample of potential end-user. *Two participants mentioned that they could not answer this question because they are not using a power wheelchair. **Two participants did not review this page and could not answer this question. ***If no response is presented, revisions were made accordingly. SCI = Spinal Cord Injury; PAG = Physical Activity Guidelines.

Supplementary files: The Canadian SCI Physical Activity Guidelines

Appendix 13: The translation of the international scientific SCI exercise guidelines to community and clinician practice guidelines

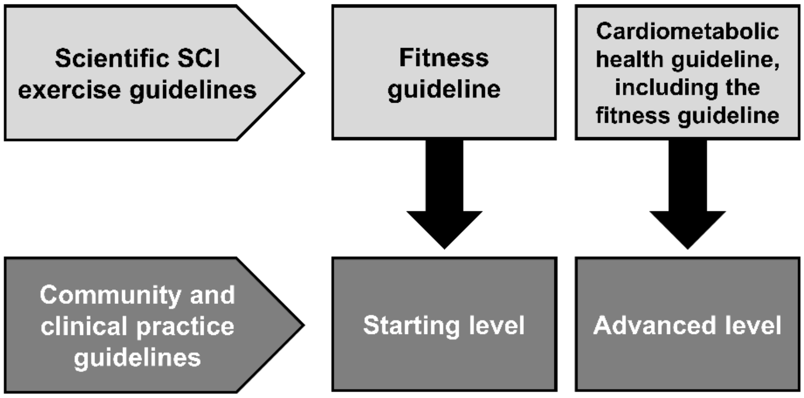

Figure A13: The translation of the international scientific SCI exercise guidelines to community and clinician practice guidelines: the example of the Canadian Spinal Cord Injury Physical Activity Guidelines.

Reference list

1. Martin Ginis, K.A., et al., *Evidence-based scientific exercise guidelines for adults with spinal cord injury: an update and a new guideline.* Spinal Cord, 2018. **56**(4): p. 308-321.
2. Arbour-Nicitopoulos, K.P., et al., *Development of an evidence-informed leisure time physical activity resource for adults with spinal cord injury: the SCI Get Fit Toolkit.* Spinal Cord, 2013. **51**(6): p. 491-500.
3. Foulon, B.L., et al., *Enhancing physical activity guidelines: a needs survey of adults with spinal cord injury and health care professionals.* Adapt Phys Activ Q, 2012. **29**(4): p. 329-45.
4. Ginis, K.A., et al., *The development of evidence-informed physical activity guidelines for adults with spinal cord injury.* Spinal Cord, 2011. **49**(11): p. 1088-96.
5. Goosey-Tolfrey, V.L., et al., *Development of scientific exercise guidelines for adults with spinal cord injury.* Br J Sports Med, 2018. **52**(18): p. 1166-1167.
6. van der Scheer, J.W., et al., *Effects of exercise on fitness and health of adults with spinal cord injury: A systematic review.* Neurology, 2017. **89**(7): p. 736-745.
7. Ginis, K.A., et al., *Leisure time physical activity in a population-based sample of people with spinal cord injury part II: activity types, intensities, and durations.* Arch Phys Med Rehabil, 2010. **91**(5): p. 729-33.
8. Ginis, K.A., et al., *Leisure time physical activity in a population-based sample of people with spinal cord injury part I: demographic and injury-related correlates.* Arch Phys Med Rehabil, 2010. **91**(5): p. 722-8.
9. Best, K.L., K.P. Arbour-Nicitopoulos, and S.N. Sweet, *Community-based physical activity and wheelchair mobility programs for individuals with spinal cord injury in Canada: Current reflections and future directions.* J Spinal Cord Med, 2017. **40**(6): p. 777-782.
10. Brawley, L.R. and A.E. Latimer, *[Physical activity guidelines for Canadians: strategies for dissemination of the message, expectations for change and evaluation].* Appl Physiol Nutr Metab, 2007. **32 Suppl 2F**: p. S189-205.
11. Latimer, A.E., L.R. Brawley, and R.L. Bassett, *A systematic review of three approaches for constructing physical activity messages: What messages work and what improvements are needed?* Int J Behav Nutr Phys Act, 2010. **7**: p. 36.
12. Brouwers, M.C., et al., *AGREE II: advancing guideline development, reporting and evaluation in health care.* CMAJ, 2010. **182**(18): p. E839-42.

13. Latimer-Cheung, A.E., et al., *Evidence-informed recommendations for constructing and disseminating messages supplementing the new Canadian Physical Activity Guidelines.* BMC Public Health, 2013. **13**: p. 419.
14. Hoekstra F, Ginis KM. Physical Activity Guidelines for Adults with Spinal Cord Injury. 2019. doi:10.17605/OSF.IO/RYP8E.
15. Hicks, A.L., et al., *The effects of exercise training on physical capacity, strength, body composition and functional performance among adults with spinal cord injury: a systematic review.* Spinal Cord, 2011. **49**(11): p. 1103-27.
16. Kawanishi, C.Y. and M. Greguol, *Physical activity, quality of life, and functional autonomy of adults with spinal cord injuries.* Adapt Phys Activ Q, 2013. **30**(4): p. 317-37.
17. Tomasone, J.R., et al., *Spinal Cord Injury, Physical Activity, and Quality of Life: A Systematic Review.* Kinesiology Review, 2013. **2**: p. 113-129.
18. Perrier, M.J., et al., *An investigation of seasonal variation in leisure-time physical activity in persons with spinal cord injury.* Spinal Cord, 2012. **50**(7): p. 507-11.
19. Perrier, M.J., et al., *Type, intensity and duration of daily physical activities performed by adults with spinal cord injury.* Spinal Cord, 2017. **55**(1): p. 64-70.
20. Fekete, C. and A. Rauch, *Correlates and determinants of physical activity in persons with spinal cord injury: A review using the International Classification of Functioning, Disability and Health as reference framework.* Disabil Health J, 2012. **5**(3): p. 140-50.
21. Williams, T.L., B. Smith, and A. Papathomas, *The barriers, benefits and facilitators of leisure time physical activity among people with spinal cord injury: a meta-synthesis of qualitative findings.* Health Psychol Rev, 2014. **8**(4): p. 404-25.
22. Best, K.L., et al., *Systematic Review and Meta-Analysis of Peer-Led Self-Management Programs for Increasing Physical Activity.* Int J Behav Med, 2016. **23**(5): p. 527-38.
23. Wolfe, D.L., et al., *Physical Activity and SCI*, in *Spinal Cord Injury, 2010, Rehabilitation Evidence. Version 3.0*, J.J. Eng, et al., Editors. 2010.
24. Martin Ginis, K.A., *Takin' it to the Streets: A Community-University Partnership Approach to Physical Activity Research and Knowledge Translation.* Kinesiology Review, 2012. **1**: p. 190-196.
25. Smith, B., et al., *Narrative as a knowledge translation tool for facilitating impact: translating physical activity knowledge to disabled people and health professionals.* Health Psychol, 2015. **34**(4): p. 303-13.
26. Martin Ginis, K.A., S. Jorgensen, and J. Stapleton, *Exercise and sport for persons with spinal cord injury.* PM R, 2012. **4**(11): p. 894-900.

27. Gainforth, H.L., et al., *Testing the feasibility of training peers with a spinal cord injury to learn and implement brief action planning to promote physical activity to people with spinal cord injury.* J Spinal Cord Med, 2015. **38**(4): p. 515-25.
28. Shirazipour, C.H., J.R. Tomasone, and K.A. Martin Ginis, *Enhancing health care professionals' and trainees' knowledge of physical activity guidelines for adults with and without SCI.* J Spinal Cord Med, 2018: p. 1-12.
29. Martin Ginis, K.A., et al., *Psychosocial factors associated with physical activity in ambulatory and manual wheelchair users with spinal cord injury: a mixed-methods study.* Disabil Rehabil, 2017. **39**(2): p. 187-192.
30. Arbour-Nicitopoulos, K.P., K.A. Ginis, and A.E. Latimer, *Planning, leisure-time physical activity, and coping self-efficacy in persons with spinal cord injury: a randomized controlled trial.* Arch Phys Med Rehabil, 2009. **90**(12): p. 2003-11.
31. Latimer, A.E., K.A. Martin Ginis, and K. Arbour, *The efficacy of an implementation intention intervention for promoting physical activity among individuals with spinal cord injury: A randomized controlled trial. .* Rehabilitation Psychology, 2006. **54**(4): p. 273-280.
32. Scelza, W.M., et al., *Perceived barriers to exercise in people with spinal cord injury.* Am J Phys Med Rehabil, 2005. **84**(8): p. 576-83.

AGREE Reporting Checklist
2016

This checklist is intended to guide the reporting of clinical practice guidelines.

CHECKLIST ITEM AND DESCRIPTION	REPORTING CRITERIA	Page #
DOMAIN 1: SCOPE AND PURPOSE		
**1. OBJECTIVES**<br>*Report the overall objective(s) of the guideline. The expected health benefits from the guideline are to be specific to the clinical problem or health topic.*	☒ Health intent(s) (i.e., prevention, screening, diagnosis, treatment, etc.)<br>☒ Expected benefit(s) or outcome(s)<br>☒ Target(s) (e.g., patient population, society)	Intro + appendix 1
**2. QUESTIONS**<br>*Report the health question(s) covered by the guideline, particularly for the key recommendations.*	☒ Target population<br>☐ Intervention(s) or exposure(s)<br>☐ Comparisons (if appropriate)<br>☐ Outcome(s)<br>☐ Health care setting or context	Intro and appendix 1
**3. POPULATION**<br>*Describe the population (i.e., patients, public, etc.) to whom the guideline is meant to apply.*	☒ Target population, sex and age<br>☒ Clinical condition (if relevant)<br>☐ Severity/stage of disease (if relevant)<br>☐ Comorbidities (if relevant)<br>☐ Excluded populations (if relevant)	Intro and appendix 1
DOMAIN 2: STAKEHOLDER INVOLVEMENT		
**4. GROUP MEMBERSHIP**<br>*Report all individuals who were involved in the development process. This may include members of the steering group, the research team involved in selecting and reviewing/rating the evidence and individuals involved in formulating the final recommendations.*	☒ Name of participant<br>☒ Discipline/content expertise (e.g., neurosurgeon, methodologist)<br>☒ Institution (e.g., St. Peter's hospital)<br>☐ Geographical location (e.g., Seattle, WA)<br>☒ A description of the member's role in the guideline development group	Methods and appendix 3
**5. TARGET POPULATION PREFERENCES AND VIEWS**<br>*Report how the views and preferences of the target population were sought/considered and what the resulting outcomes were.*	☒ Statement of type of strategy used to capture patients'/publics' views and preferences (e.g., participation in the guideline development group, literature review of values and preferences)<br>☒ Methods by which preferences and views were sought (e.g., evidence from literature, surveys, focus groups)<br>☒ Outcomes/information gathered on patient/public information<br>☒ How the information gathered was used to inform the guideline development process and/or formation of the recommendations	Methods + Figure 1 + Appendix 1 + OSF.
**6. TARGET USERS**<br>*Report the target (or intended) users of the guideline.*	☒ The intended guideline audience (e.g. specialists, family physicians, patients, clinical or institutional leaders/administrators)	Intro and appendix 2

	☐ How the guideline may be used by its target audience (e.g., to inform clinical decisions, to inform policy, to inform standards of care)	
DOMAIN 3: RIGOUR OF DEVELOPMENT		
**7. SEARCH METHODS**<br>*Report details of the strategy used to search for evidence.*	☐ Named electronic database(s) or evidence source(s) where the search was performed (e.g., MEDLINE, EMBASE, PsychINFO, CINAHL)<br>☐ Time periods searched (e.g., January 1, 2004 to March 31, 2008)<br>☐ Search terms used (e.g., text words, indexing terms, subheadings)<br>☐ Full search strategy included (e.g., possibly located in appendix)	Methods
**8. EVIDENCE SELECTION CRITERIA**<br>*Report the criteria used to select (i.e., include and exclude) the evidence. Provide rationale, where appropriate.*	☒ Target population (patient, public, etc.) characteristics<br>☒ Study design<br>☐ Comparisons (if relevant)<br>☒ Outcomes<br>☐ Language (if relevant)<br>☒ Context (if relevant)	Methods and Appendix 4
**9. STRENGTHS & LIMITATIONS OF THE EVIDENCE**<br>*Describe the strengths and limitations of the evidence. Consider from the perspective of the individual studies and the body of evidence aggregated across all the studies. Tools exist that can facilitate the reporting of this concept.*	☒ Study design(s) included in body of evidence<br>☒ Study methodology limitations (sampling, blinding, allocation concealment, analytical methods)<br>☒ Appropriateness/relevance of primary and secondary outcomes considered<br>☐ Consistency of results across studies<br>☐ Direction of results across studies<br>☐ Magnitude of benefit versus magnitude of harm<br>☒ Applicability to practice context	Methods
**10. FORMULATION OF RECOMMENDATIONS**<br>*Describe the methods used to formulate the recommendations and how final decisions were reached. Specify any areas of disagreement and the methods used to resolve them.*	☒ Recommendation development process (e.g., steps used in modified Delphi technique, voting procedures that were considered)<br>☒ Outcomes of the recommendation development process (e.g., extent to which consensus was reached using modified Delphi technique, outcome of voting procedures)<br>☒ How the process influenced the recommendations (e.g., results of Delphi technique influence final recommendation, alignment with recommendations and the final vote)	Methods + appendix 5-8 + Figure 1
**11. CONSIDERATION OF BENEFITS AND HARMS**<br>*Report the health benefits, side effects, and risks that were considered when formulating the recommendations.*	☒ Supporting data and report of benefits<br>☒ Supporting data and report of harms/side effects/risks<br>☐ Reporting of the balance/trade-off between benefits and harms/side effects/risks<br>☐ Recommendations reflect considerations of both benefits and harms/side effects/risks	Table 1 and 2; Appendix 7
**12. LINK BETWEEN RECOMMENDATIONS AND EVIDENCE**	☒ How the guideline development group linked and used the evidence to inform recommendations	Appendix 6 and 7

Describe the explicit link between the recommendations and the evidence on which they are based.	☒ Link between each recommendation and key evidence (text description and/or reference list)<br>☒ Link between recommendations and evidence summaries and/or evidence tables in the results section of the guideline	
**13. EXTERNAL REVIEW**<br>*Report the methodology used to conduct the external review.*	☒ Purpose and intent of the external review (e.g., to improve quality, gather feedback on draft recommendations, assess applicability and feasibility, disseminate evidence)<br>☒ Methods taken to undertake the external review (e.g., rating scale, open-ended questions)<br>☒ Description of the external reviewers (e.g., number, type of reviewers, affiliations)<br>☒ Outcomes/information gathered from the external review (e.g., summary of key findings)<br>☒ How the information gathered was used to inform the guideline development process and/or formation of the recommendations (e.g., guideline panel considered results of review in forming final recommendations)	Table 2 and append ices;
**14. UPDATING PROCEDURE**<br>*Describe the procedure for updating the guideline.*	☒ A statement that the guideline will be updated<br>☐ Explicit time interval or explicit criteria to guide decisions about when an update will occur<br>☐ Methodology for the updating procedure	Appen dix 1
DOMAIN 4: CLARITY OF PRESENTATION		
**15. SPECIFIC AND UNAMBIGUOUS RECOMMENDATIONS**<br>*Describe which options are appropriate in which situations and in which population groups, as informed by the body of evidence.*	☒ A statement of the recommended action<br>☒ Intent or purpose of the recommended action (e.g., to improve quality of life, to decrease side effects)<br>☒ Relevant population (e.g., patients, public)<br>☐ Caveats or qualifying statements, if relevant (e.g., patients or conditions for whom the recommendations would not apply)<br>☐ If there is uncertainty about the best care option(s), the uncertainty should be stated in the guideline	Table 1-2 + append ix 1
**16. MANAGEMENT OPTIONS**<br>*Describe the different options for managing the condition or health issue.*	☐ Description of management options<br>☐ Population or clinical situation most appropriate to each option	Appen dix 1 + Figure 2
**17. IDENTIFIABLE KEY RECOMMENDATIONS**<br>*Present the key recommendations so that they are easy to identify.*	☒ Recommendations in a summarized box, typed in bold, underlined, or presented as flow charts or algorithms<br>☐ Specific recommendations grouped together in one section	Figure 2 + appen dix 1
DOMAIN 5: APPLICABILITY		
**18. FACILITATORS AND BARRIERS TO APPLICATION**<br>*Describe the facilitators and barriers to the guideline's application.*	☒ Types of facilitators and barriers that were considered<br>☐ Methods by which information regarding the facilitators and barriers to implementing recommendations were sought (e.g., feedback from key stakeholders, pilot testing of guidelines before widespread implementation)	Discus sion and Appen dix 1

	☐ Information/description of the types of facilitators and barriers that emerged from the inquiry (e.g., practitioners have the skills to deliver the recommended care, sufficient equipment is not available to ensure all eligible members of the population receive mammography)<br>☐ How the information influenced the guideline development process and/or formation of the recommendations	
**19. IMPLEMENTATION ADVICE/TOOLS**<br>*Provide advice and/or tools on how the recommendations can be applied in practice.*	☒ Additional materials to support the implementation of the guideline in practice. For example:<br>○ Guideline summary documents<br>○ Links to check lists, algorithms<br>○ Links to how-to manuals<br>○ Solutions linked to barrier analysis (see Item 18)<br>○ Tools to capitalize on guideline facilitators (see Item 18)<br>○ Outcome of pilot test and lessons learned	Discusi on
**20. RESOURCE IMPLICATIONS**<br>*Describe any potential resource implications of applying the recommendations.*	☐ Types of cost information that were considered (e.g., economic evaluations, drug acquisition costs)<br>☐ Methods by which the cost information was sought (e.g., a health economist was part of the guideline development panel, use of health technology assessments for specific drugs, etc.)<br>☐ Information/description of the cost information that emerged from the inquiry (e.g., specific drug acquisition costs per treatment course)<br>☐ How the information gathered was used to inform the guideline development process and/or formation of the recommendations	Discus sion
**21. MONITORING/ AUDITING CRITERIA**<br>*Provide monitoring and/or auditing criteria to measure the application of guideline recommendations.*	☐ Criteria to assess guideline implementation or adherence to recommendations<br>☐ Criteria for assessing impact of implementing the recommendations<br>☒ Advice on the frequency and interval of measurement<br>☐ Operational definitions of how the criteria should be measured	Discu ssion
DOMAIN 6: EDITORIAL INDEPENDENCE		
**22. FUNDING BODY**<br>*Report the funding body's influence on the content of the guideline.*	☒ The name of the funding body or source of funding (or explicit statement of no funding)<br>☒ A statement that the funding body did not influence the content of the guideline	Appe ndix 1
**23. COMPETING INTERESTS**<br>*Provide an explicit statement that all group members have declared whether they have any competing interests.*	☐ Types of competing interests considered<br>☐ Methods by which potential competing interests were sought<br>☐ A description of the competing interests<br>☐ How the competing interests influenced the	Appe ndix 1

	guideline process and development of recommendations	

From:
Brouwers MC, Kerkvliet K, Spithoff K, on behalf of the AGREE Next Steps Consortium. The AGREE Reporting Checklist: a tool to improve reporting of clinical practice guidelines. *BMJ* 2016;352:i1152. doi: 10.1136/bmj.i1152.

For more information about the AGREE Reporting Checklist, please visit the AGREE Enterprise website at http://www.agreetrust.org.
